# High-Throughput Shotgun Metagenomics of Microbial Footprints Uncovers a Cocktail of Noxious Antibiotic Resistance Genes in the Winam Gulf of Lake Victoria, Kenya

**DOI:** 10.1155/jotm/7857069

**Published:** 2024-12-23

**Authors:** Sandra Khatiebi, Kelvin Kiprotich, Zedekiah Onyando, John Mwaura, Clabe Wekesa, Celestine N. Chi, Chrispinus Mulambalah, Patrick Okoth

**Affiliations:** ^1^Department of Biological Sciences, School of Natural and Applied Sciences, Masinde Muliro University of Science and Technology, P.O. Box 190, Kakamega 50100, Kenya; ^2^Department of Soil Sciences, Faculty of Agrisciences, Stellenbosch University, Private Bag X1, Matieland, Stellenbosch 7602, South Africa; ^3^Department of Biochemistry, Max Planck Institute for Chemical Ecology, Jena 8 07745, Germany; ^4^Department of Medical Biochemistry and Microbiology, Uppsala University, P.O. Box 582751 23, Uppsala, Sweden; ^5^Department of Medical Microbiology and Parasitology, School of Medicine, Masinde Muliro University of Science and Technology, P.O. Box 190, Kakamega 50100, Kenya

**Keywords:** antibiotic resistance genes (ARGs), KEGG pathways, pollutants, shotgun metagenomics

## Abstract

**Background:** A diverse range of pollutants, including heavy metals, agrochemicals, pharmaceutical residues, illicit drugs, personal care products, and other anthropogenic contaminants, pose a significant threat to aquatic ecosystems. The Winam Gulf of Lake Victoria, heavily impacted by surrounding human activities, faces potential contamination from these pollutants. However, studies exploring the presence of antibiotic resistance genes (ARGs) in the lake remain limited. In the current study, a shotgun metagenomics approach was employed to identify ARGs and related pathways. Genomic DNA was extracted from water and sediment samples and sequenced using the high-throughput Illumina NovaSeq platform. Additionally, phenotypic antibiotic resistance was assessed using the disk diffusion method with commonly used antibiotics.

**Results:** The analysis of metagenomes sequences from the Gulf ecosystem and Comprehensive Antibiotic Resistance Database (CARD) revealed worrying levels of ARGs in the lake. The study reported nine ARGs from the 37 high-risk resistant gene families previously documented by the World Health Organization (WHO). *Proteobacteria* had the highest relative abundance of antibiotic resistance (53%), *Bacteriodes* (4%), *Verrucomicrobia* (2%), *Planctomycetes Chloroflexi*, *Firmicutes* (2%), and other unclassified bacteria (39%). Genes that target protection, replacement, change, and antibiotic-resistant efflux were listed in order of dominance. Kyoto Encyclopedia of Genes and Genomes (KEGG) pathway analysis revealed antibiotic resistance to beta-lactamase and vancomycin. Phenotypic resistance to vancomycin, tetracycline, sulfamethoxazole, erythromycin, trimethoprim, tetracycline, and penicillin was reported through the zone of inhibition.

**Conclusions:** This study highlights that the Winam Gulf of Lake Victoria in Kenya harbors a diverse array of antibiotic-resistant genes, including those conferring multidrug resistance. These findings suggest that the Gulf could be serving as a reservoir for more antibiotic-resistant genes, posing potential risks to both human health and aquatic biodiversity. The insights gained from this research can guide policy development for managing antibiotic resistance in Kenya.

## 1. Introduction

Global freshwater pollution is a growing public health burden. Multiclass pollutants including heavy metals, agrochemicals, pharmaceutical residues, drugs of abuse, and other anthropogenic pollutants collectively constitute a public health burden with calamitous consequences on aquatic biodiversity [1]. Land use practices and the unauthorized discharge of industrial effluent, including municipal and sewage wastes, bear the burden of Lake Victoria's pollution [2]. Some studies have reported evidence of harm caused by pollution in the lake [3]. For example, one study found high levels of heavy metals such as lead, cadmium, and mercury in the water, which can have negative effects on the health of aquatic organisms and humans who consume fish from the lake [4]. Additionally, another study noted an increase in algal blooms in the lake, which can deplete oxygen levels and harm fish populations [5].

Antibiotics, and agrochemical and heavy metal residues from pharmaceutical products may alter the microbial assemblages of an aquatic environment, causing the expression of genes for antibiotic resistance [6]. Residents depend on Lake Victoria for the primary supply of drinking water and food chain; thus, its quality has an immediate impact on their health [7]. Surface water has reportedly been found to have a variety of antimicrobial resistance (AMR) genes [8]. The acquisition of environmental AMR genes through horizontal gene transfer is a potential outcome of pollution [9, 10]. Numerous studies have shown that diverse aquatic environments across the world contain antibiotic-resistant genes [11–14]. In order to reduce the hazards of antibiotic resistance in both humans and animals, a long-term solution must be sought. Aquatic environments operate as a turning point for the environmental release, dissemination, and persistence of antimicrobial-resistant genes [15]. Emerging evidence suggests that the discharge from healthcare facilities, landfills, wastewater treatment facilities, anthropogenic compartments, and pharmaceutical factories considerably increases the amount of antimicrobial-resistant genes in aquatic environments [16]. Numerous studies have shown that urban water systems contain antibiotic-resistant genes, putting the health of city dwellers in danger [17]. Urban freshwater lakes provide water for home and industrial usage, farming, swimming and fishing, and other leisure pursuits. It is difficult to evaluate the effects on human and animal health and the risks associated with the rise of antibiotic-resistant genes in urban water environments [18]. Due to increased anthropogenic pollution and fecal contamination of urban freshwater lakes brought by dense populations, aquatic bacteria are now more likely to develop antibiotic-resistant genes [19].

The majority of antibiotics and their metabolites are excreted in urine and feces, and improper drug disposal exacerbates this problem [20]. Due to their deleterious consequences on environmental species and ecosystems, antibiotics' presence in the environment is a cause for worry [21]. Through ingestion in food chains, antibiotic bioaccumulation in aquatic fauna and flora indirectly jeopardizes human health [22]. The spread and establishment of antibiotic-resistant genes are made worse by the chemical toxicity of antibiotics in water systems [23]. Failure of treatment plants and widespread occurrences in the environment, including, water, air, farms, and soils, is probably the cause of the emergence of antibiotic-resistant genes [24]. Pollution may make it easier for pathogens to horizontally transferred genes for multidrug resistance, causing a heavy burden on healthcare systems [25]. Antimicrobial-resistant genes are also cross-selectively enhanced by other water contaminants such as heavy metals and personal care products [26]. The presence of resistant genes compromises the ability to treat infections [27]. However, the use of antibiotic-resistant genes by terrorists to achieve a nefarious outcome in genetically modified organisms is a growing concern worldwide [28]. Antibiotic-resistant genes are easier to find and characterize, thanks to the Comprehensive Antibiotic Research Database (CARD) database. Based on their resistance profiles and closeness in sequence, resistant genes are further divided into the following categories: antibiotic inactivation, antibiotic target alteration, and antibiotic efflux [29]. Antibiotic resistance profile and mechanism include the extensive information annotated for each gene [30].

Numerous studies on the pharmaceuticals have been conducted, including the ones on antibiotics in the greater Lake Victoria Basin [31]. Previous studies found that a significant amount of antibiotics was being released from the Nyalenda Kisumu sewage lagoons [32]. According to a study done in the northern region of Lake Victoria in Uganda, the Nakivubo wetland served as a horizontal gene pool and exchange platform for disease-resistant genes affected by heavy metals [33]. The Winam Gulf (Kisumu), a smaller portion of the greater Lake Victoria, has little documented history on antibiotic resistance [34]. WHO first identified AMR as a global public health threat in the year 2014, and the report highlighted the need for a coordinated global response to combat (AMR), including improving surveillance and monitoring, promoting the rational use of antimicrobials, and investing in research to develop new antimicrobial agents [35].

Shotgun metagenomics is a novel technique for analyzing antibiotic-resistant genes in aquatic ecosystems [36]. The method has several advantages as it allows for the detection of a wide range of antibiotic-resistant genes including those that are not well characterized or previously unknown in polluted water and sediments [1, 10]. The approach is not limited to target genes but rather providing a comprehensive view of the entire microbial community in the sample [37]. Shotgun metagenomics also provides information on the pathways and genetic context of the antibiotic-resistant genes, including mobile genetic elements like plasmids [38]. This information is pertinent to the understanding of the mechanism of antibiotic resistance and the potential for horizontal gene transfer between different microbial species [39]. Furthermore, shotgun metagenomics approach can be used to monitor the changes in the abundance and diversity of antibiotic resistance genes (ARGs) over time [40, 41]. This information can provide insight into the impact of anthropogenic activities on the spread and persistence of antibiotic-resistant genes in aquatic ecosystems [42]. Several studies have shown shotgun metagenomics approaches providing functional microbial profiles, occurrence ARG gene pathways, and the host bacteria [43]. Previous studies have reported the occurrence and distribution of antibiotics-resistant genes in aquatic ecosystems [17].

Rapid advancements in high-throughput sequencing (HTS) technologies and machine learning approaches produce large volumes of genomic and metagenomics sequences, enabling highly accurate classification of microbial communities across various ecosystems [44]. The goal of this study was to employ a shotgun metagenomics sequencing approach to determine antimicrobial susceptibility and to investigate the phenotypic and genotypic AMR pathways among bacterial assemblages in the aquatic ecosystem of the Winam Gulf of Lake Victoria, Kenya.

## 2. Methodology

### 2.1. Description of the Study Area

**Figure 1:**
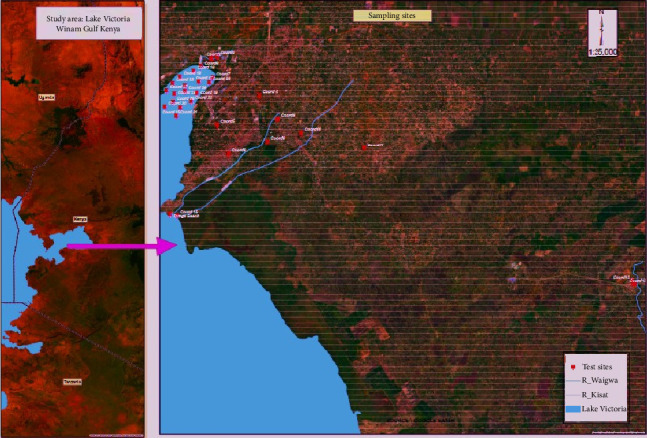
Location of Winam Gulf in Lake Victoria Kenya and GPS location of the sampling sites.

Winam Gulf is an extension of Western Kenya's border and Uganda in the Northeastern portion of Lake Victoria. It encompasses the counties of Kisumu, Homabay, Migori, Busia, and Siaya. It is a shallow inlet, measuring 35 miles (56 km) long by 15 miles broad, and it is joined to the main lake by a 3-mile channel (Figure 1). The Winam Gulf has an annual precipitation range of more than 1800 mm in higher elevations in the eastern parts. Winam Gulf is located at latitude 0° 14′ 14.40″ N and longitude 34° 34′ 28.79″ E. The region experiences about 1966 mm of annual rainfall on average, and the average annual temperature is 23.1°C (Kisumu Weather & Climate & Weather by Month—Climate-Data.). According to the Kenya Population and Housing Census (KPHC) 2019, the region is home to 397,957 people, small-scale agricultural retail markets, fisheries, and industries such as tourism, food processing, oil refining, plastics, furniture, and cement. Additionally, it has been noted for having a variety of retail establishments, including supermarkets, and educational institutions with a large student body, including RIAT, Kisumu National Polytechnic, Maseno University, and the Great Lakes. The Kenya Medical Research Institute (KEMRI), Jaramogi Oginga Odinga Teaching and County Referral Hospital, and other privately owned hospitals and clinics are among the other important establishments.

### 2.2. Sampling and Processing of the Samples

The Kisumu wastewater treatment plant's (WWTP) effluent discharges into the lake. Kisumu industrial effluent, fish-landing beaches, stormwater entrance points, Kisumu Water and Sewerage Company (KIWASCO) treatment facility, rivers Kisat, Wigwa, Nyamasaria, Nyando, and lake locations were all sampled. Sediment and water samples were purposefully sampled from 15 different sites in the Winam Gulf, Kisumu. The specific coordinates of the sites (Supporting Information). This sampling approach was selected to ensure a diverse range of organic and inorganic pollutants. Water samples were collected in sterile plastic bottles (500 mL), sealed, and transported to the laboratory in cooler boxes at 4°C within 12 h. These samples were then stored at −80°C for subsequent metagenomics analysis. Sediment samples were collected by scooping a 0–2-cm layer from each site and placed in sterile bottles. A total of 89 water and sediment samples were collected, cleaned with nitric acid, rinsed with distilled water, and transported to the laboratory for further processing.

A homogenized sample consisting of water and sediment from all sampling sites (130 samples in total) was created by pooling and thoroughly mixing the individual samples. This mixture was then sieved using grade 1 filter papers (Whatman) to remove any large particles and debris. A subsample (10 mL) was taken from a 20-L sample, centrifuged (5000 × *g* for 10 min), and the supernatant decanted. This process was repeated for the entire homogenized sample. The sediment cell debris was resuspended by distilled water, vortexed, and transferred into 2-mL Eppendorf tubes for further processing.

### 2.3. Physicochemical Analyses of the Sampling Sites

Salinity, total dissolved solids (TDSs), pH, temperature, conductivity, and dissolved oxygen were measured. Water temperature was measured with a thermometer before the samples were taken, and the results were recorded before computing pH values. The pH meter was calibrated using standard buffers of 4.0, 7.0, and 10.0 to assure its accuracy. Each reading was recorded after 4 mL of each sample had been collected, and the rod was cleaned before taking the next pH reading. The conductivity, which gauges how well a solution conducts electricity, was tested using a conductivity meter.

After calibration using a standard solution and the results were recorded, the conductivity probe was dipped directly into the water samples to evaluate the electrical conductivity. Salinity was estimated using a refractometer by first calculating the refractive index of a small water sample that was placed on the device's prism. A conversion table had been created, and it was used to convert the refractive index to salinity. A turbidimeter was used to test the turbidity of a water sample that was placed inside a cuvette. By counting the amount of light reflected off the suspended particles in the samples, the apparatus calculated the turbidity value.

Water samples (5 mL) were evaporated in a preweighed container to determine the TDSs before weighing the residual solids. Following the heating of the samples to evaporate the water, the container was once more weighed to ascertain the TDS content. The weight of the dried solids was divided by the volume of the original sample to calculate TDS, with the findings being represented in milligrams per liter. A dissolved oxygen meter was used to measure the concentrations of dissolved oxygen. The equipment assessed the partial pressure of oxygen dissolved in the solution, while the probe was submerged directly into the water samples. The measurements were made as the concentration of dissolved oxygen.

### 2.4. Phenotypic Characterization

The phenotypic characteristics were determined in the wet laboratory to isolate bacteria from water and sediment samples. The culture procedure involved serial dilutions of samples of pooled water and sediment, used to isolate the bacteria. Thereafter, the samples were cultured in nutrient broth and incubated for 24 h at 37°C. The grown cultures were subcultured on MacConkey and nutrient agar for colony differentiation and later subcultured to obtain pure cultures. Gram staining technique was carried out for further identification of the bacteria.

### 2.5. Evaluation of Antibiotic Resistance

The following drugs were tested on five pure isolates using Kirby–Bauer disk diffusion method [45] as a confirmatory test to support metagenomic analysis: Ampicillin (10 μg), penicillin (10 μg), imipenem (10 μg), vancomycin (30 μg), ceftazidime (30 μg), gentamicin (120 μg), tazobactam (110 μg), erythromycin (15 μg), tetracycline (30 μg), cefoxitin (30 μg), ceftriaxone (30 μg), sulfamethoxazole (25 μg), and trimethoprim (25 μg). The analysis was evaluated ensuring that the initial bacterial concentration adhered to the recommended 0.5 McFarland standard (1.5 × 10^8^ CFU/mL) to maintain the integrity of the results. The formation of zones of inhibition was considered susceptible to the drugs, while those that did not form the zone of inhibition were considered resistant to the drugs.

### 2.6. Genomic DNA Extraction and Shotgun Metagenomics Sequencing

The isolation of genomic DNA from the sample was done in a previous study [46]. Briefly, the sample in a 2-mL Eppendorf tube was lysed with 2% of 0.7 mL CTAB buffer containing 20 mM EDTA, 0.1 M Tris–HCl pH 8.0, 1.4 M NaCl, and 2% CTAB. Beta-mercaptoethanol at a concentration of 0.4% was added and incubated at 65°C for 45 min with gentle mixing by inversion every 15 minutes. The mixture was then subjected to chloroform–isoamyl extraction and centrifugation. Cold isopropanol was used to precipitate the DNA, which was then washed with ethanol and dried overnight. The DNA pellet was resuspended in TE buffer and stored at −20°C for shotgun metagenomics. The quality and concentration of the DNA sample were assessed using agarose gel electrophoresis and a Qubit 2.0 Fluorometer, respectively (Thermo Fisher Scientific). Sequencing was done by metagenomic DNA library fragmentation, end repair and A-tailing, adapter ligation, and PCR amplification on an Illumina NovaSeq platform to obtain reads (Novogene (UK) Company Ltd).

### 2.7. Metagenomics Analyses

Assembled metagenome sequence reads were matched with MicroNR reference database. A library of taxonomically informative gene families and abundance was determined. The scaftig length was used in MetaGeneMark to predict genes, and CD-HIT was used to obtain the gene catalog for each sample while maintaining a 95% clustering threshold. DIAMOND software (V0.9.9.110) was used to annotate species and align UniGene sequences with those of bacteria, fungi, viruses, and archaea that were taken from the NCBI's NR database. The process of functional annotation involved inferring sequence similarity with KEGG, eggNOG, and CAZy databases, while the functional category hit distribution was annotated through MG-RAST subsystem categorization.

## 3. Results

### 3.1. Physicochemical Analysis

Eighty-nine water samples from the lake, nearby rivers, Nyamasaria, Kisat, and Wigwa, and WWTPs were used to provide the physicochemical statistical data for this study. Physicochemical parameters play a significant role in determining microbial profiles in water samples. These parameters include factors such as pH, dissolved oxygen levels, turbidity salinity, and TDSs (Table 1). The values pH, salinity, TDS, EC, and COD were within acceptable WHO standards, whereas turbidity was above the WHO acceptable standards.

### 3.2. Rarefaction and Antibiotic Resistance Gene Annotations

**Figure 2:**
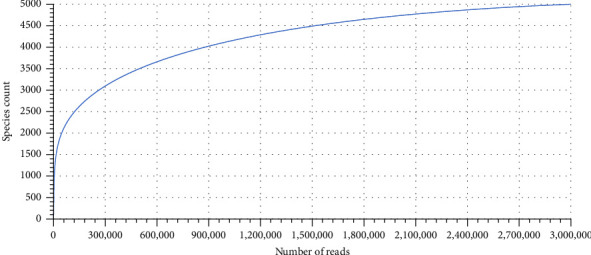
Rarefaction curve indicating the annotated number of species against the obtained metagenome sequence reads.

The dataset for sample 1[S1] metagenomes contained 2,889,335 sequences totaling 726,434,355 base pairs with an average length of 251 bps. Out of the sequences tested, 10,983 sequences (0.38%) failed to pass the quality control (QC) pipeline. Furthermore, dereplication identified 0 sequences as artificial duplicate reads. Of the sequences that passed QC, 19,423 sequences (1%) contain ribosomal RNA genes while 2,316,422 sequences (80.48%) contain predicted proteins with known functions, and 542,507 sequences (18.85%) contained predicted proteins with unknown functions. The rarefaction curve indicated that if a reasonable number of species individuals is further sampled above 5000, the curve would flatten to the right indicating a reliability of obtained metagenome sequence reads (Figure 2).

**Figure 3:**
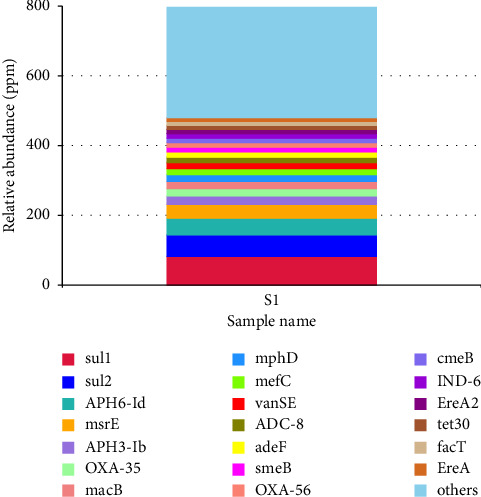
Relative abundance of resistance genes in the Winam Gulf, Lake Victoria. S1 (pooled metagenomic sample libraries).

The ARG genes were identified against the CARD [47] using BLASTP with an e-value threshold of ≤ 1e − 5 to ensure the identification of homologous sequences with high confidence. Relative abundance of different resistance genes was calculated, and the abundance table of resistant genes was determined. A total of 21 antibiotic-resistant genes were detected in the sediments and water samples taken from the Winam Gulf of Lake Kenya. The genes *sul1*, *sul2*, *APH6*-*1d*, *msrE*, and *APH3*-*1b* dominated (Figure 3).

### 3.3. Antibiotic-Resistant Genes per Bacteria Phyla

**Figure 4:**
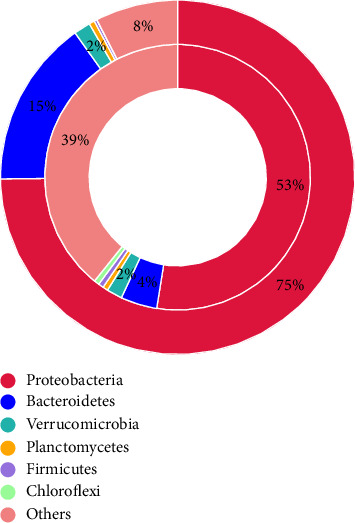
Percentage abundance of bacteria phyla and percentage of antibiotic-resistant genes per phyla in the Winam Gulf, Lake Victoria. Outer circle—abundance of bacteria phyla. Inner circle—abundance of AGRs per bacteria phyla.

Antibiotic-resistant genes are genes that confer resistance to antibiotics and are found in many different bacteria phyla in aquatic ecosystems. Here is a brief representation of the main phyla and their associated antibiotic-resistant genes in the aquatic Winam Gulf Kisumu (Figure 3). The identified Phyla were *Proteobacteria*, *Bacteroidetes*, *Verrucomicrobia Planctomycetes*, *Firmicutes*, *Chloroflexi,* and others. The percentages of the phyla were as follows: *Proteobacteria*, 75%, *Bacteriodes*, 15%, *Verrucomicrobia* 2%, and others 8%; *Planctomycetes*, *Firmicutes*, *and Chloroflexi* were less than 1%. The relative percentage abundance of antibiotic-resistant genes presents in each phylum: *Proteobacteria 53%*, *Bacteroidetes 4%*, and others *39%*; *Verrucomicrobia 2%*, and *Planctomycetes*, *Firmicutes*, and *Chloroflexi* remaining 2% (Figure 4).

### 3.4. Mode of Action Mechanisms of Antibiotic-Resistant Genes

**Figure 5:**
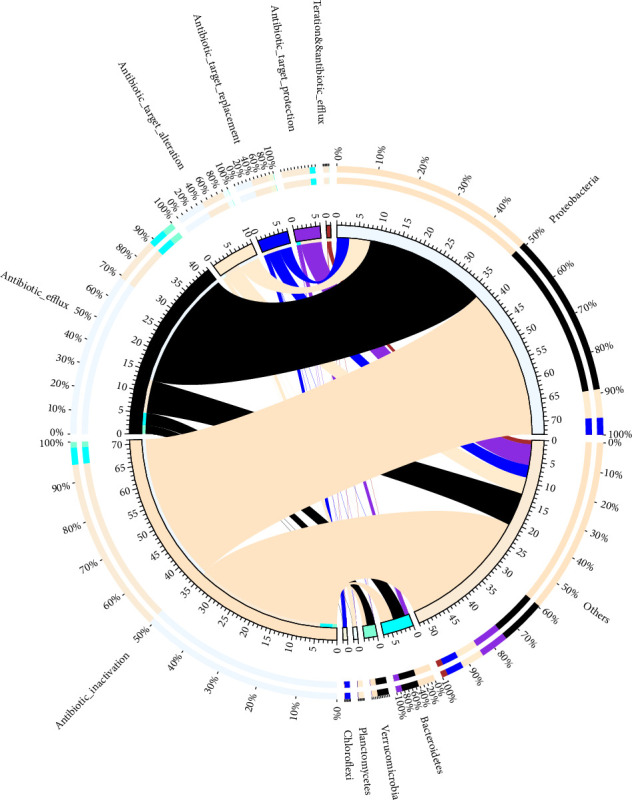
A circle plot illustrating the association between microbial phyla and antibiotic resistance mechanisms in the metagenomic dataset. The outer circle is divided into two sections: the left section represents different antibiotic resistance mechanisms, such as efflux pumps, enzymatic degradation, and target modification, with each mechanism labeled and color-coded, while the right section depicts microbial phyla, including *Proteobacteria*, *Firmicutes*, and *Bacteroidetes*, with their respective percentage abundances indicated alongside their segments. Chords connect microbial phyla to the resistance mechanisms they are associated with, with the thickness of each chord reflecting the strength of the association, such as the number of resistant genes linked.

The association between microbial phyla and AMR mechanisms and mode of action in the metagenomic dataset was analyzed. Antibiotic efflux, antibiotic inactivation, antibiotic target change, antibiotic target replacement, and antibiotic target protection were demonstrated with the predicted ARGs. Antibiotic efflux was shown by *Proteobacteria*, *Bactrioides*, and *Verrucomicrobia*. Antibiotic inactivation was present in *Proteobacteria*, *Bactrioides*, *Verrucomicrobia*, and *Planctomycetes*. Antibiotic targets changes *Proteobacteria*, *Bacteroidetes*, *Verrucomicrobia Planctomycetes*, and *Chloroflexi*. Antibiotic target replacement and antibiotic target protection were seen in all the phyla present (Figure 5).

### 3.5. Phenotypic Antibiotic Resistance Patterns

**Figure 6:**
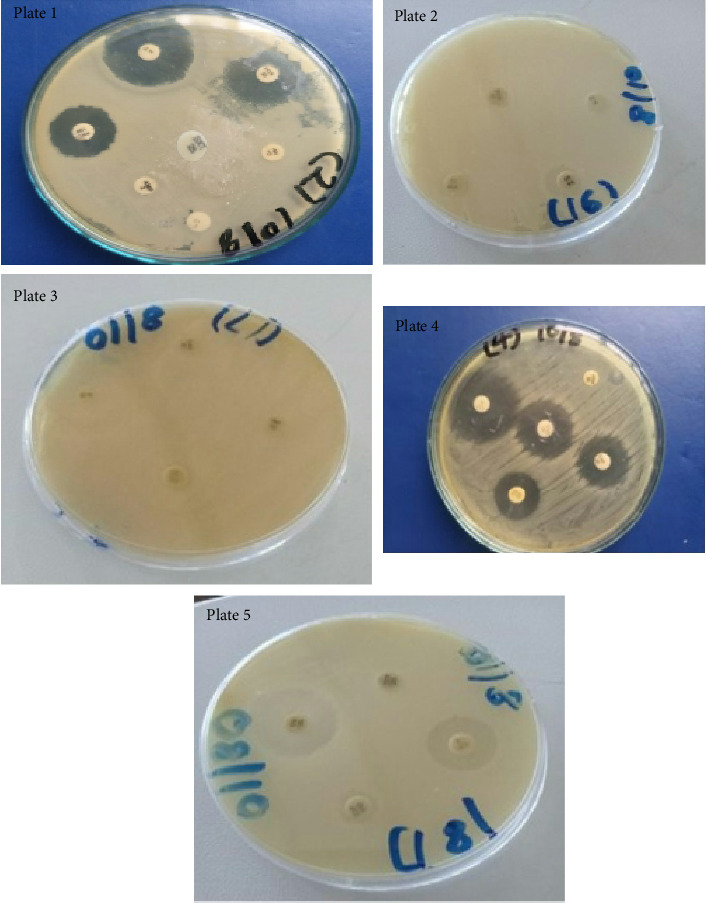
Antibiotic resistance patterns of Gram-negative and Gram-positive bacteria. Plate 1—Gram-negative bacteria, Plate 2—Gram-positive bacteria, Plate 3—Gram-positive bacteria, Plate 4—Gram-negative bacteria, and Plate 5—Gram-negative bacteria.

The results of the current study revealed valuable information on susceptibility patterns of bacteria on different antibiotics. Antibiotic resistance was observed in the commonly used antibiotics (Figure 6). Plate 1—Gram-negative bacteria showed resistance to ampicillin, penicillin, imipenem, and sulfamethoxazole; Plate 2—Gram-positive bacteria exhibited resistance in piperacillin, meropenem, and vancomycin; Plate 3—Gram-negative resistance was observed in imipenem, tetracycline, ceftazidime, and erythromycin; Plate 5—Gram-negative showed resistance to piperacillin/tazobactam and trimethoprim; and Plate 4 showed resistance in tetracycline.

### 3.6. Pathways Analysis

In this study, the KEGG pathway gave insights into the mechanism of antibiotic resistance and the genes responsible for specific resistance patterns.

#### 3.6.1. Beta-Lactamase Resistance Pathway

**Figure 7:**
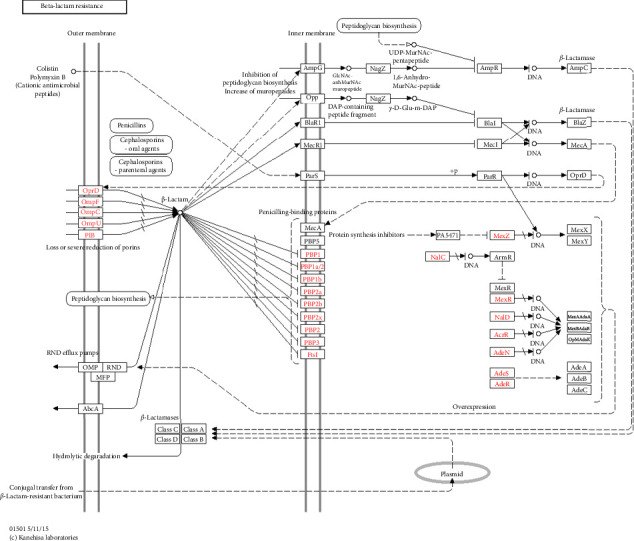
Pathway showing beta-lactamase antimicrobial resistance. OprD; resistance mechanism decreases the production of OprD porin, which is involved in the entry of carbapenem antibiotics like imipenem (Imipenem repression of porin) ompF (outer membrane pore protein F). Causes multidrug resistance repression of porin OmpF. OmpC—outer membrane pore protein. OmpU—outer membrane protein OmpU. PIB is the major outer membrane protein. All four are broken by hydrolytic degradation in the intermembrane space, thus not getting to the penicillin-binding protein and pumped out. Plasmid transfers the resistant genes by MerCR and thus induces the methicillin drug resistance. Mexz: confers multidrug resistance. Ades ABC is an overexpression efflux pump and is responsible for acquiring fluoroquinolone resistance. Inactivation of Aden thus diminishes and increases the antibiotic resistance.

Beta-lactamase is an enzyme produced by certain bacteria to confer resistance to beta-lactamase antibiotics such as penicillin, cephalosporin, and carbapenem. The KEGG pathway below includes the genes that encode for beta-lactamase and the genes involved in resistance mechanisms. The pathway begins with the biosynthesis of beta-lactamase antibiotics, which are degraded by beta-lactamase enzymes. The genes that encode for these enzymes are carried on mobile genetic elements (plasmids), which are transferred between bacteria to spread the antibiotic resistance traits (Figure 7).

#### 3.6.2. Vancomycin Resistance Pathway

**Figure 8:**
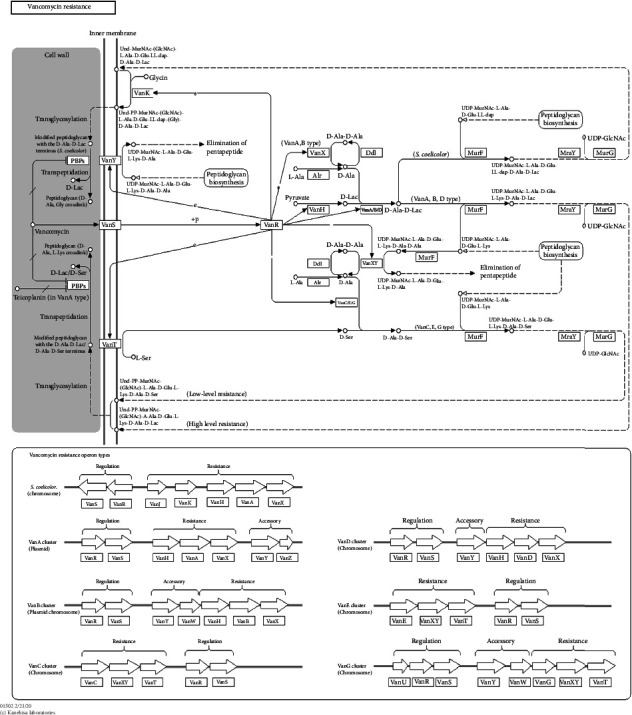
Pathway showing VanS and VanR regulate the expression of vancomycin resistance, while VanX reprograms cell wall biosynthesis to produce peptidoglycan chain precursors. Van A and B conferred acquired resistance, carried on by plasmids.

The KEGG pathway below shows the genetic and biochemical involved in the resistance to vancomycin. This pathway reveals how the cell wall peptidoglycan is modified by D-ala terminus to prevent the effective binding of the drug, thus vancomycin resistance. In addition, the pathway shows the different types of vancomycin resistance operons by the production of enzymes that modify the structure of vancomycin such as VanA and VanB, thus reducing its ability to bind to the target site (Figure 8).

## 4. Discussion

Antibiotic-resistant genes are a growing public health concern in polluted aquatic ecosystems. These ecosystems are often contaminated with antibiotics, which has led to selection spread of bacteria carrying ARGs [48]. The horizontal transfer of genetic material between bacteria often facilitates the spread of ARGs in aquatic ecosystems [49]. These genes have the potential to proliferate quickly and can be transferred between bacterial species in aquatic ecosystems, which could result in widespread antibiotic resistance among the populations [50, 51]. Since the species of bacteria can survive even when exposed to high levels of antibiotics, research has shown that antibiotic-resistant genes can have a significant impact on aquatic bacterial flora and alter microbial assemblages. Additionally, the spread of antibiotic-resistant genes to fish and plants can cause additional harm down the food chain [52]. Understanding antibiotic-resistant genes in aquatic environments using advanced metagenomics can help inform policy development and prevention of antibiotic resistance [53, 54].

Physicochemical parameters such as pH, TDS, EC, COD, and turbidity play crucial roles in assessing water quality and its impacts on microbial profiles [55]. In the study, the values of pH, TDS, EC, and COD were within the acceptable WHO standards, whereas the turbidity was above WHO acceptable standards. This is consistent with the findings of a previous study on Lake Victoria [56]. The elevated turbidity indicates a potential pollution issue in the Winam Gulf part of Lake Victoria and is consistent with previous studies [57]. The increased turbidity can disrupt the microbial profiles by altering light availability and nutrient dynamics in freshwater lake ecosystems [58]. Increased turbidity can also enhance the acquisition of antibiotic-resistant genes among microbial communities [59]. It creates favorable conditions for horizontal gene transfer mechanism such as conjugation, transformation, and transduction to take place more frequently [60]. This highlights the interconnectedness between environmental factors and antibiotic resistance in natural ecosystems [61].

The current study reported 21 antibiotic-resistant genes consistent with the findings of a previous study, which were practically identical in quantity to the 26 antibiotic-resistant genes discovered in the aquatic ecosystem of Lake Lonar Soda in India [62]. The study reported antibiotic-resistant genes to aminoglycosides and sulfonamides, consistent with the findings from other investigations [17]. This is consistent with research conducted in freshwater environments where Sulfonamide 1 was the major ARGS followed by Sulfonamide 2 [17]. Long-term antibiotic pollution in aquatic ecosystems may be responsible for the presence of nine ARGs from the 37 WHO identified high-risk AMR gene families, which has led to the coevolution of environmental chemicals and microbial genomes [63].

*Proteobacteria was* the dominant phyla with antibiotic-resistant genes, followed by *Bacteroidetes*, *Verrucomicrobia*, *Planctomycetes*, *Chloroflexi,* and *Firmicutes* [64]. *Proteobacteria* have a high ARGS abundance, which is related to their flexible metabolism and capacity for environmental adaptation [65]. *Proteobacteria* is a diverse group of bacteria commonly found in aquatic environments [66]. Studies documented that *Proteobacteria* possess high levels of antibiotic-resistant genes that enable them to survive in environments with antibiotics [67]. Aquatic environments such as lakes and rivers receive large amounts of antibiotic residues from human and animal waste [68]. This constant exposure to antibiotics has provided selective pressure that has favored the survival and proliferation of antibiotic resistance in *Proteobacteria* [69]. *Proteobacteria* often live-in close association with other microorganisms such as algae and protozoa, thus allowing interspecies spread of antibiotic-resistant genes [70, 71]. The high level of antibiotic-resistant genes of *Proteobacteria* in aquatic ecosystems is a public health burden as they may contribute to the spread of antibiotic resistance in clinical and environmental setups [72]. Examples of human and animal pathogenic *Proteobacteria* found in aquatic ecosystem have been documented to possess antibiotic-resistant genes: *Vibrio cholerae*, which inhabit rivers, lakes, and coastal waters, have been shown to possess genes that confer antibiotic resistance to multiple antibiotics [73]. *Pseudomonas aeruginosa*, a common opportunistic pathogen that infects humans and animals often found in aquatic environments, has been shown to possess a variety of antibiotic-resistant genes [74]. *Proteobacteria* found in aquatic ecosystems, including *Escherichia coli*, *Salmonella enterica,* and *Klebsiella pneumoniae*, pathogenic to humans and animals possess genes that confer resistance to multiple antibiotics [75].

Due to its several antibiotic resistance mechanisms, *Bacteroidetes*, a common gut flora, has been reported to have multidrug ARGs [76]. Bacteriodes possess ARGs in aquatic ecosystems due to their great capacity to evolve and develop new ways to survive in different environments [77]. The findings of antibiotic resistance of this phylum provide insight into understanding the mechanisms of antibiotic resistance. *Bacteroidetes* have the ability to quickly produce and spread antibiotic-resistant genes in response to changing environments, which allows them to continue to thrive despite the presence of antibiotics [78]. Examples of *Bacteroidetes* species that cause diseases in humans and animals are *Bacteroidetes vulgatus*, *B. thetaiotaomicron*, and *Clostridium* difficile are some of the most noxious human pathogens with demonstrable antibiotic resistance to multiple antibiotics, thus a public health concern [79]. *Verrucomicrobia* , a Gram-negative bacterium, has been shown to have ARGs in water sediments [80]. A few species of this phylum have been isolated from fresh seawater, soils, and human feces [81]. Both aquatic and terrestrial *Planctomycetes* can be found, with *Chloroflexi* inhabiting deep freshwater lakes and *Firmicutes* occupying a variety of environments, including commensals on skin surfaces [82]. The Winam Gulf revealed the least antibiotic-resistant genes in *Planctomycetes*, *Chloroflexi*, and *Firmicutes*, which is consistent with earlier research on aquatic environments [83]. These microbes possess the least antibiotic-resistant genes compared to other bacterial groups because they have evolved other mechanisms to survive in their environment that do not involve antibiotic resistance [84]. *Planctomycetes* have been shown to have the ability to metabolize complex organic compounds, such as proteins, lipids, and polysaccharides, which may provide them with a competitive advantage over other bacteria [85]. Additionally, *Chloroflexi* and *Firmicutes* have been shown to be highly adaptable to changing environmental conditions, thus contributing to the ability to survive in all habitats [86].

The genes for additional, unidentified antibiotic resistance were also very abundant; this may be attributed to mutation brought about by the severe circumstances induced by multiple classes of contaminants [87]. The genetic diversity of free-living microbes has made natural habitats, which have become hotspots for resistant mechanisms, a source of antibiotic-resistant genes in addition to harmful bacteria [79]. As a defense strategy, aquatic bacteria naturally produce ARGs. These genes can be multiclass pollutant-mediated, which is prevalent in aquatic ecosystems. The Winam Gulf's main antibiotic resistance mechanism was antibiotic efflux, followed by drug inactivation and target modification, which agrees with the findings of a previous study conducted [88]. Antibiotic-resistant genes that can develop because of chronic exposure to multiple classes of contaminants, such as pharmaceuticals, agrochemicals, and heavy metals, may also be present in the antibiotic resistance genome [89]. Examples of a study conducted in Baltic Sea found that antibiotic-resistant genes found in bacterial community were acquired due to human-derived pollutants [90]. Another study conducted in the Mediterranean Sea found that antibiotic-resistant genes were present in bacterial communities of the sea and were likely acquired through chronic exposure to pharmaceuticals, heavy metals, and other pollutants [12]. Due to the efflux pump's capacity to identify a variety of substrates expressed by several pathogens, antibiotic efflux is the most preferred method of acquiring bacterial resistance [65]. Numerous Gram-negative biofilm bacteria, particularly aquatic biofilm, were reported to have the antibiotic resistance efflux mechanism [91]. The antibiotic efflux was first identified in the tetracycline resistance of *E*. *coli*, as well as in the multidrug resistance efflux [92]. Bacteria that produce B-lactamases, aminoglycoside enzymes, and chloramphenicol acetyltransferases have been found to exhibit antibiotic inactivation resistance [93]. Resistance to penicillin, cephalosporins, carbapenem, amoxil, and erythromycin, among other antibiotics, has been linked to antibiotic inactivation [94]. Small modifications to the target molecules and entire bacterial cells have been used to alter antibiotic targets in bacteria, thereby preventing the antibiotics from reaching the intended target [95]. Selection in the presence of the antibiotic and spontaneous mutation of the bacterial gene on the chromosome causes the target position to alter [96]. Antimicrobial drugs work to block a variety of biological structures, including the cell wall, cell membrane, protein synthesis, nucleic synthesis, and chemicals involved in biological metabolism [97]. Pathogens are increasingly modifying antibiotics, such as glycopeptide and polymyxin antibiotics, by chemically altering the cell wall components. The least frequent responses were antibiotic target replacement, antibiotic target protection, antibiotic target modification, and efflux, which was consistent with earlier studies on aquatic environments [77]. Antibiotic resistance in microorganisms has long been demonstrated using phenotypic antibiotic resistance patterns [99]. Gram-positive and Gram-negative bacteria were exposed to a variety of drugs; some were sensitive, whereas others displayed the antibiotic resistance patterns [100, 101]. The subsequent antibiotics showed the signs of antibiotic resistance: In both Gram-negative and Gram-positive bacteria, clindamycin is a broad-spectrum antibiotic that prevents early chain elongation by interfering with protein synthesis [101]. Tetracycline prevents bacterial growth by interfering with protein synthesis and damaging the cell membrane. It is broad-spectrum for both Gram-negative and Gram-positive modes of action. A broad-spectrum antibiotic called sulfamethoxazole prevents bacterial growth by blocking the manufacture of nucleic acids and proteins by inhibiting the sequential step in bacterial folic acid [102]. Vancomycin resistance in enterococci is a very serious threat to humans with few treatment options being available [103]. Vancomycin works on Gram-positive bacteria by blocking the second step of cell wall formation, which prevents bacterial development [104]. Erythromycin belongs to the macrolide class and works by targeting the bacterial ribosomes by blocking the elongation of peptide chains, thus slowing the bacteria growth. Erythromycin is a broad-spectrum bacterium for both Gram-negative and Gram-positive bacteria [105]. Erythromycin is widely used in treating bacterial respiratory tract infection, soft skin infection, and sexually transmitted infection; thus, its resistance is a threat to human health [106]. Trimethoprim mode of action inhibits the synthesis of folic acid, thus inhibiting bacterial growth and replication [107]. Trimethoprim is effective in the treatment of respiratory tract infection, urinary tract infection, and gastrointestinal tract infection; thus, its resistance is a threat to human health [108]. Pollution is a potential source of resistance for glycopeptide-producing organisms in the presence of genes encoding homologous VanA, VanR, VanS, VanR, VanH, and VanX [109]. A key development in our understanding of the molecular and genetic basis of vancomycin resistance is the discovery of the vancomycin-resistant pathway in the bacterial community in the Winam Gulf. This highlights the importance of studying the bacterial communities and their interactions in the spread of antibiotic resistance and, thus, developing new strategies to combat antibiotic resistance. Beta-lactamase resistance in Gram-positive bacteria primarily occurs because of the alteration of penicillin-binding proteins with enzymatic degradation as a minor pathway [110]. This study revealed OprD, OmpF, OmpC, OmpU, and PIB altered by hydrolytic degradation blocking them from binding to penicillin-binding proteins, leading to the resistance of nondulation division efflux pump. This mechanism eliminates numerous antibiotic Classes A, B, C, and D from inside bacterial cells, which contributes to multidrug resistance [111]. In Gram-negative bacteria, RND efflux pumps offer intrinsic antibiotic resistance with the potential for cross-resistance to several drugs. The most common resistance mechanism is lactamases, and the synergy created by the interaction of these several mechanisms is crucial in deciding how resistance will ultimately manifest itself phenotypically [112]. Ceftazidime has a broad spectrum of activity against both Gram-positive and Gram-negative bacteria [113]. Imipenem, a member of the carbapenem family, is a broad-spectrum inhibitor of Gram-positive and Gram-negative bacteria's ability to produce cell walls [94]. A bactericidal, broad-spectrum antibiotic called piperacillin suppresses the formation of cell walls [114]. The genotypic profiles of antibiotic resistance agree with other investigations and support the phenotypic antibiotic resistance patterns that were reported in previous studies as well as in bioremediation to regulate pathogenic bacteria [115, 116].

## 5. Conclusion

This study brings to the fore novel findings that shed light on the extent and diversity of ARGs in the polluted aquatic ecosystem of the Winam Gulf of Lake Victoria, Kenya. *Proteobacteria*, the major phyla that inhabit aquatic ecosystems, showed higher genes of antibiotic resistance. This suggests that the spread of antibiotic-resistant genes is not limited to human pathogens and the wild nonpathogenic bacteria can contribute to the spread of ARGs. The discovery of ARGs in the environmental bacteria due to the continuous pollution of multiclass pollutants of pharmaceuticals, drugs of abuse, personal care products, agrochemicals, and heavy metals adds connectors. This is a novel that highlights the complexity of the spread of antibiotic resistance in polluted aquatic ecosystems. These findings underscore the need for continued monitoring and research to understand the mechanisms, extent of antibiotic resistance in this ecosystem, and the steps to reduce their spread. Additionally, this study recommends further investigation into the effects of physicochemical parameters of the Winam Gulf on the distribution and abundance of antibiotics resistance. The discovery of ARGs in polluted aquatic ecosystems is a threat to human health and environment and is relevant to SDG 3 and SDG 6. Addressing the spread of antibiotic resistance in this ecosystem is important for achieving these SDGs, ensuring a healthy and sustainable future for all. The presence of multiclass pollutants capable of transmitting and incorporating genes for antibiotic resistance among the microbes in the Winam Gulf Kisumu confirms that the Lake is not only a reservoir but also a medium of evolution and spread of antibiotic resistance. The presence of ARGs in polluted aquatic ecosystems is a major concern in polluted aquatic ecosystems and public health as it is difficult to treat infections.

## Figures and Tables

**Table 1:** Mean and standard values of physicochemical parameters of water in the Winam Gulf of Lake Victoria and WHO standards.

Sampling site	*N*	pH	Salinity	Turbidity	TDS	EC	COD
Kiwasco water	89	8.50 ± 0.23	0.11 ± 0.04	6.93 ± 2.02	184.42 ± 2.74	368.45 ± 11.60	520.60 ± 39.40
Nyando water	89	7.06 ± 0.05	0.10 ± 0.01	7.29 ± 1.29	182.87 ± 7.02	365.21 ± 3.27	381.50 ± 4.92
Kisat water	89	7.04 ± 0.09	0.04 ± 0.02	6.67 ± 1.28	299.20 ± 1.36	604.00 ± 4.17	272.33 ± 52.54
Lake water	89	7.07 ± 0.38	0.02 ± 0.01	7.12 ± 0.17	41.9 ± 1.46	84.43 ± 0.67	321.00 ± 6.76
Wigwa water	89	7.20 ± 0.12	0.05 ± 0.03	9.74 ± 1.44	284.13 ± 3.18	588.47 ± 5.49	284.00 ± 7.74
Nyamasaria water	89	7.66 ± 0.07	0.21 ± 0.04	11.06 ± 1.54	266.53 ± 28.66	535.84 ± 11.40	397.25 ± 6.64
WHO STDS		6.5–8.5	> 0.5 ppt	> 5NTU	> 500	> 1000S/m	< 5 mg/O_2_l

*Note:* There was a significant difference between the physicochemical parameters in various water samples from different sites (*F* = (*p* < 0.01)).

## Data Availability

The molecular dataset generated during the current study are available in the NCBI GenBank with Accession No. PRJNA992979. Other data are available in this manuscript.

## Supporting Information

The supporting information contains the coordinate list of the sampling sites (Supporting Information).
