# Supplementary material for: High-Throughput Shotgun Metagenomics of Microbial Footprints Uncovers a Cocktail of Noxious Antibiotic Resistance Genes in the Winam Gulf of Lake Victoria, Kenya
Source: J Trop Med. 2024 Dec 23;2024:7857069. doi: 10.1155/jotm/7857069 (PMC11685326; doi:10.1155/jotm/7857069)
Supplement: Supporting Information — Additional supporting information can be found online in the Supporting Information section. [file 7857069.f1.pdf]

	Site Name	Easting	Northing	Location
	Coord 1	695021.44	9990768.83	On Kisat River
	Coord 2	695041.95	9990805.53	On Kisat River
	Coord 3	695292.15	9990885.55	On Kisat River
	Coord 4	697142.34	9989179.06	Stormwater
	Coord 5	695293.26	9987900.89	Stormwater
	Coord 6	694588.07	9990444.19	In Lake Victoria
	Coord 7	695101.12	9989950.29	In Lake Victoria
	Coord 8	697948.19	9988145.57	On Waigwa River
	Coord 9	697495.98	9987143.58	Kiwasco Outlet
	Coord 10	699004.77	9987553.38	Stormwater
	Coord 11	701749.26	9986846.43	Stormwater
	Coord 12	713574.33	9980978.86	On Nyando River
	Coord 13	713691.33	9980855.95	On Nyando River
	Coord 14	707222.92	9966758.02	On Nyando River
	Coord 15	693520.54	9983875.81	On Nyamasaria River
	Coord 16	694262.00	9990245.00	In Lake Victoria
	Coord 17	694490.00	9989777.00	In Lake Victoria
	Coord 18	693660.00	9989999.00	In Lake Victoria
	Coord 19	694399.00	9989296.00	In Lake Victoria
	Coord 20	694953.00	9989745.00	In Lake Victoria
	Coord 21	693878.00	9989606.00	In Lake Victoria
	Coord 22	694154.00	9988933.00	In Lake Victoria
	Coord 23	693338.00	9989711.00	In Lake Victoria
	Coord 24	693415.00	9989258.00	In Lake Victoria
	Coord 25	693677.00	9988651.00	In Lake Victoria
	Coord 26	693367.00	9988909.00	In Lake Victoria
	Coord 27	693072.00	9989401.00	In Lake Victoria
	Coord 28	693885.00	9989355.00	In Lake Victoria
	Coord 29	693367.00	9988909.00	In Lake Victoria
	Coord 30	693004.00	9988677.00	In Lake Victoria
	Coord 31	693499.00	9988294.00	In Lake Victoria
